# Supplementary material for: Maternal characteristics and their relation to early mother-child interaction and cognitive development in toddlers
Source: PLoS One. 2025 Jan 15;20(1):e0301876. doi: 10.1371/journal.pone.0301876 (PMC11734904; doi:10.1371/journal.pone.0301876)
Supplement: S1 Table — (DOCX) [file pone.0301876.s001.docx]

**S1 Table.** Demographics characteristics of both samples.

| Sample | Infants (n = 38) | Toddlers (n = 72) |
| --- | --- | --- |
| Maternal age | 31.34 (SD = 4.58); 17-45 | 31.63 (SD = 5.13); 21-44 |
| Nationality | Austria: 31  Germany: 1  Other: 2  Not specified: 4 | Austria: 40  Germany: 15  Other: 5  Not specified: 12 |
| Education | No professional training: 1  Apprenticeship: 3  Professional/technical college/seminary: 1  Higher education entrance qualification: 11  University: 18  Not specified: 4 | No professional training: 2  Apprenticeship: 10  Professional/technical college/seminary: 3  Higher education entrance qualification: 23  University: 27  Not specified: 9 |
| Parity | First child: 27  Second child: 5  Third child: 2  Not specified: 4 | First child: 37  Second child: 25  Third child: 2  Not specified: 8 |
| Relationship status | Partnership: 32  Single parenting: 2  Not specified: 4 | Partnership: 58  Single parenting: 2  Not specified: 12 |
| Work status | Maternal leave: 27  Marginal/part-time: 6  housewife: 1  Not specified: 12 | Maternal leave: 35  Marginal/part-time: 22  Self-employed: 3  Student: 1  Not specified: 12 |
