# Supplementary material for: Maternal characteristics and their relation to early mother-child interaction and cognitive development in toddlers
Source: PLoS One. 2025 Jan 15;20(1):e0301876. doi: 10.1371/journal.pone.0301876 (PMC11734904; doi:10.1371/journal.pone.0301876)
Supplement: S2 Table — (DOCX) [file pone.0301876.s002.docx]

**S2 Table.** Intraclass correlation coefficients (ICC) for the CARE-Index subscales for infants (*n* = 11) and toddlers (*n* = 23).

| Subscale | Toddler |
| --- | --- |
| 1. Dyadic Synch. | 0.79 |
| 2. Sensitive | 0.85 |
| 3. Controlling | 0.79 |
| 4. Unresponsive | 0.79 |
| 5. Cooperative | 0.78 |
| 6. Compulsive | 0.25 |
| 7. Threat. coercive | 0.86 |
| 8. Disarm. coercive. | 0.65 |

| Subscale | Infant |
| --- | --- |
| 1. Dyadic Synch. | 0.87 |
| 2. Sensitive | 0.90 |
| 3. Controlling | 0.96 |
| 4. Unresponsive | 0.95 |
| 5. Cooperative | 0.92 |
| 6. Compulsive | 0.87 |
| 7. Difficult | 0.77 |
| 8. Passive. | 0.91 |
