# Supplementary material for: Maternal characteristics and their relation to early mother-child interaction and cognitive development in toddlers
Source: PLoS One. 2025 Jan 15;20(1):e0301876. doi: 10.1371/journal.pone.0301876 (PMC11734904; doi:10.1371/journal.pone.0301876)
Supplement: S3 Table — (DOCX) [file pone.0301876.s003.docx]

**S3 Table.** CARE-Index interaction quality: Means, standard deviations, and spearman’s correlations for infants (*n* = 38) and toddlers (*n* = 72).

|  | *Infant* | | | | | | | | | |
| --- | --- | --- | --- | --- | --- | --- | --- | --- | --- | --- |
| Variable | *M* | *SD* | 1 | 2 | 3 | 4 | 5 | 6 | 7 | 8 |
| 1.Mean age | 11.55 | 2.37 |  |  |  |  |  |  |  |  |
| 2. Dyadic Synch. | 7.39 | 2.21 | .26 |  |  |  |  |  |  |  |
| 3. Sensitive | 7.47 | 2.27 | .18 | .97** |  |  |  |  |  |  |
| 4. Controlling | 2.82 | 2.56 | -.06 | -.34* | -.35* |  |  |  |  |  |
| 5. Unresponsive | 3.71 | 2.72 | -.07 | -.45** | -.46** | -.64** |  |  |  |  |
| 6. Cooperative | 7.18 | 2.22 | .25 | .95** | .91** | -.26 | -.48** |  |  |  |
| 7. Compulsive | 1.55 | 2.97 | .19 | -.44** | -.46** | .50** | -.11 | -.41* |  |  |
| 8. Difficult | 2.97 | 2.16 | -.30 | .17 | .21 | .09 | -.26 | .21 | -.51** |  |
| 9. Passive | 2.29 | 2.75 | -.05 | -.25 | -.25 | -.47** | .68** | -.34* | -.40* | -.29 |

|  | *Toddler* | | | | | | | | | |
| --- | --- | --- | --- | --- | --- | --- | --- | --- | --- | --- |
| Variable | *M* | *SD* | 1 | 2 | 3 | 4 | 5 | 6 | 7 | 8 |
| 1.Mean age | 21.85 | 4.46 |  |  |  |  |  |  |  |  |
| 2. Dyadic Synch. | 7.90 | 2.18 | .07 |  |  |  |  |  |  |  |
| 3. Sensitive | 8.00 | 2.21 | .07 | .96** |  |  |  |  |  |  |
| 4. Controlling | 2.60 | 2.27 | .02 | -.45** | -.44** |  |  |  |  |  |
| 5. Unresponsive | 3.39 | 2.29 | .01 | -.39** | -.43** | -.52** |  |  |  |  |
| 6. Cooperative | 7.92 | 2.15 | .07 | .98** | .94** | -.44** | -.38** |  |  |  |
| 7. Compulsive | 1.33 | 2.42 | .01 | -.32** | -.31** | .19 | .05 | -.32** |  |  |
| 8. Threat. coercive | 2.50 | 1.85 | -.15 | -.16 | -.09 | .08 | .11 | -.12 | -.57** |  |
| 9. Disarm. coercive | 2.25 | 1.64 | .02 | -.29* | -.37** | .20 | .17 | -.36** | -.25* | -.09 |

*Note.* *M* and *SD* are used to represent mean and standard deviation, respectively. * indicates *p* < .05. ** indicates *p* < .01. Note that in both samples, the mean dyadic synchrony scores may be categorised “adequate” (7-10).
