# Supplementary material for: Maternal characteristics and their relation to early mother-child interaction and cognitive development in toddlers
Source: PLoS One. 2025 Jan 15;20(1):e0301876. doi: 10.1371/journal.pone.0301876 (PMC11734904; doi:10.1371/journal.pone.0301876)
Supplement: S4 Table — (DOCX) [file pone.0301876.s004.docx]

**S4 Table**. Maternal personality traits (BFI-10) and mother-child interaction: Means, standard deviations, and spearman’s correlations split for infants (*n* = 34) and toddlers (*n* = 61).

|  | *Infant* | | | | | | |
| --- | --- | --- | --- | --- | --- | --- | --- |
| Variable | *M* | *SD* | 1 | 2 | 3 | 4 | 5 |
| 1. Neuroticism | 2.96 | 0.92 |  |  |  |  |  |
| 2. Extraversion | 3.62 | 0.88 | -.27 |  |  |  |  |
| 3. Openness | 3.47 | 1.04 | .23 | .05 |  |  |  |
| 4. Agreeableness | 3.1 | 0.70 | -.41+ | .24 | .21 |  |  |
| 5. Conscientiousness | 3.54 | 0.89 | -.13 | -.09 | -.01 | .07 |  |
| 6. Dyadic Synch. | 7.35 | 2.28 | -.04 | .07 | .13 | .10 | .13 |
| 7. Sensitive | 7.41 | 2.34 | -.10 | .16 | .08 | .16 | .11 |
| 8. Controlling | 3.00 | 2.62 | .06 | .03 | -.17 | -.04 | .29 |
| 9. Unresponsive | 3.59 | 2.73 | -.01 | -.16 | .05 | -.10 | -.41+ |
| 10. Cooperative | 7.15 | 2.27 | -.04 | .08 | .16 | .12 | .10 |
| 11. Compulsive | 1.74 | 3.09 | .23 | .12 | -.16 | -.30 | .21 |
| 12. Difficult | 3.06 | 2.24 | .05 | -.18 | -.09 | .02 | -.25 |
| 13. Passive | 2.06 | 2.66 | -.20 | -.29 | .06 | .06 | -.18 |

|  | *Toddler* | | | | | | |
| --- | --- | --- | --- | --- | --- | --- | --- |
| Variable | *M* | *SD* | 1 | 2 | 3 | 4 | 5 |
| 1. Neuroticism | 2.82 | 0.86 |  |  |  |  |  |
| 2. Extraversion | 3.71 | 0.92 | -.24 |  |  |  |  |
| 3. Openness | 3.67 | 0.86 | -.14 | .22 |  |  |  |
| 4. Agreeableness | 3.37 | 0.63 | .04 | .10 | .15 |  |  |
| 5. Conscientiousness | 3.89 | 0.74 | .14 | .09 | .08 | .12 |  |
| 6. Dyadic Synch. | 7.84 | 2.11 | -.05 | .08 | .13 | .21 | -.01 |
| 7. Sensitive | 7.93 | 2.15 | -.02 | .07 | .12 | .18 | -.03 |
| 8. Controlling | 2.57 | 2.20 | .13 | .20 | -.23 | -.10 | .09 |
| 9. Unresponsive | 3.48 | 2.29 | -.09 | -.23 | .15 | -.11 | -.05 |
| 10. Cooperative | 7.92 | 2.07 | -.01 | .06 | .09 | .19 | -.03 |
| 11. Compulsive | 1.30 | 2.45 | .31+ | -.08 | -.09 | .09 | .06 |
| 12. Threat. coercive | 2.46 | 1.79 | .01 | .15 | .01 | -.12 | -.17 |
| 13. Disarm. coercive | 2.33 | 1.69 | -.26 | -.13 | .05 | -.13 | -.01 |

*Note.* *M* and *SD* are used to represent mean and standard deviation, respectively. + indicates *p_corrected_* < .10.
