# Supplementary material for: Maternal characteristics and their relation to early mother-child interaction and cognitive development in toddlers
Source: PLoS One. 2025 Jan 15;20(1):e0301876. doi: 10.1371/journal.pone.0301876 (PMC11734904; doi:10.1371/journal.pone.0301876)
Supplement: S5 Table — (A) Maternal depression (BDI-II) and mother-child interaction: Means, standard deviations, and spearman’s correlations in toddlers (n = 62). (B) Maternal experience of emotions (SEE) and mother-child interaction: Means, standard deviations, and spearman’s correlations for infants (n = 25). (DOCX) [file pone.0301876.s005.docx]

**S5 Table.** (A) Maternal depression (BDI-II) and mother-child interaction: Means, standard deviations, and spearman’s correlations in toddlers (*n* = 62). (B) Maternal experience of emotions (SEE) and mother-child interaction: Means, standard deviations, and spearman’s correlations for infants (*n* = 25).

**A**

| Variable | *M* | *SD* | 1 |
| --- | --- | --- | --- |
| 1. BDI | 6.53 | 4.37 |  |
| 2. Dyadic Synch. | 7.82 | 2.09 | -.04 |
| 3. Sensitive | 7.94 | 2.13 | -.06 |
| 4. Controlling | 2.53 | 2.21 | .25+ |
| 5. Unresponsive | 3.52 | 2.30 | -.15 |
| 6. Cooperative | 7.90 | 2.05 | -.05 |
| 7. Compulsive | 1.27 | 2.43 | -.01 |
| 8. Threat. coercive | 2.53 | 1.87 | .15 |
| 9. Disarm. coercive | 2.29 | 1.70 | .07 |

*Note.* *M* and *SD* are used to represent mean and standard deviation, respectively. * indicates *p* < .05. + indicates *p* < .10

**B**

| Variable | *M* | *SD* | 1 | 2 | 3 | 4 | 5 | 6 | 7 |
| --- | --- | --- | --- | --- | --- | --- | --- | --- | --- |
| 1. Acceptance | 51.04 | 10.31 |  |  |  |  |  |  |  |
| 2. Overflow | 48.84 | 11.10 | -.64** |  |  |  |  |  |  |
| 3. Lack | 48.00 | 11.27 | -.52+ | .49+ |  |  |  |  |  |
| 4. Somatization | 47.48 | 9.22 | -.02 | .08 | -.13 |  |  |  |  |
| 5. Imagination | 46.64 | 8.57 | -.28 | .35 | .02 | -.02 |  |  |  |
| 6. Regulation | 56.64 | 8.85 | .63** | -.37 | -.40 | .33 | -.29 |  |  |
| 7. Self-Control | 52.84 | 11.29 | .42 | -.34 | -.38 | -.17 | -.20 | .42 |  |
| 8. Dyadic Synch. | 7.32 | 2.27 | -.23 | .16 | -.16 | .46+ | .21 | .24 | -.05 |
| 9. Sensitive | 7.28 | 2.25 | -.14 | .11 | -.18 | .44 | .07 | .34 | .08 |
| 10. Controlling | 3.20 | 2.65 | .09 | -.02 | .22 | .09 | -.02 | -.11 | .06 |
| 11. Unresponsive | 3.52 | 3.03 | .09 | -.06 | -.04 | -.33 | .02 | -.09 | -.14 |
| 12. Cooperative | 7.16 | 2.21 | -.13 | .07 | -.22 | .47+ | .28 | .35 | .13 |
| 13. Compulsive | 1.16 | 2.49 | -.04 | .19 | .11 | .30 | .04 | -.28 | -.29 |
| 14. Difficult | 3.24 | 2.40 | .14 | -.05 | -.01 | -.22 | -.13 | .22 | .40 |
| 15. Passive | 2.44 | 2.95 | .06 | -.17 | .06 | -.29 | -.10 | -.03 | -.18 |

*Note.* *M* and *SD* are used to represent mean and standard deviation, respectively. + indicates *p_corrected_* < .10. * indicates *p_corrected_* < .05. ** indicates *p_corrected_* < .01. *Acceptance* of own emotions; Experienced emotional *overflow*; Experienced *lack* of emotions; body-related symbolisation of emotions (*somatization*); imaginative symbolisation of emotions (i*magination*); Experience of emotion *regulation*; Experience of *self-control*
