# Supplementary material for: Maternal characteristics and their relation to early mother-child interaction and cognitive development in toddlers
Source: PLoS One. 2025 Jan 15;20(1):e0301876. doi: 10.1371/journal.pone.0301876 (PMC11734904; doi:10.1371/journal.pone.0301876)
Supplement: S6 Table — (DOCX) [file pone.0301876.s006.docx]

**S6 Table.** Maternal self-concept (MSWS) and mother-child interaction: Means, standard deviations, and spearman’s correlations for infants (*n* = 34) and toddlers (*n* = 61).

|  | *Infants* | | | |  | | *Toddler* | | |
| --- | --- | --- | --- | --- | --- | --- | --- | --- | --- |
| Variable | *M* | *SD* | 1 |  | | *M* | | *SD* | 1 |
| 1. MSWS Global | 52.47 | 10.31 |  |  | | 53.80 | | 8.34 |  |
| 2. Dyadic Synch. | 7.35 | 2.28 | -.01 |  | | 7.82 | | 2.11 | .23 |
| 3. Sensitive | 7.41 | 2.34 | .07 |  | | 7.93 | | 2.15 | .23 |
| 4. Controlling | 3.00 | 2.62 | .03 |  | | 2.52 | | 2.23 | .01 |
| 5. Unresponsive | 3.59 | 2.73 | -.10 |  | | 3.52 | | 2.31 | -.22 |
| 6. Cooperative | 7.15 | 2.27 | -.01 |  | | 7.90 | | 2.07 | .19 |
| 7. Compulsive | 1.74 | 3.09 | .10 |  | | 1.30 | | 2.45 | -.24 |
| 8. Difficult | 3.06 | 2.24 | -.36+ |  | | 2.52 | | 1.89 | .01 |
| 9. Passive | 2.06 | 2.66 | .03 |  | | 2.28 | | 1.71 | -.09 |

*Note.* *M* and *SD* are used to represent mean and standard deviation, respectively. + indicates *p_corrected_* < .10.
