# Supplementary material for: Maternal characteristics and their relation to early mother-child interaction and cognitive development in toddlers
Source: PLoS One. 2025 Jan 15;20(1):e0301876. doi: 10.1371/journal.pone.0301876 (PMC11734904; doi:10.1371/journal.pone.0301876)
Supplement: S7 Table — (DOCX) [file pone.0301876.s007.docx]

**S7 Table.** Child cognitive development (Bayley) and maternal factors: Means, standard deviations, and correlations with confidence intervals in the toddler sample (*n* = 41).

| Variable | *M* | *SD* | 1 |
| --- | --- | --- | --- |
| 1. Bayley | 11.83 | 2.95 |  |
| 2. BFI Neuroticism | 2.83 | 0.91 | -.12 |
| 3. BFI Extraversion | 3.72 | 1.02 | .07 |
| 4. BFI Openness | 3.74 | 0.76 | .14 |
| 5. BFI Agreeableness | 3.35 | 0.68 | .39* |
| 6. BFI Conscientiousness | 3.94 | 0.66 | .13 |
| 7. MSWS Global | 52.61 | 8.06 | .31 |

*Note.* *M* and *SD* are used to represent mean and standard deviation, respectively. * indicates *p_corrected_* < .05.
